# Sodium Channel Gene Variants in Fetuses with Abnormal Sonographic Findings: Expanding the Prenatal Phenotypic Spectrum of Sodium Channelopathies

**DOI:** 10.3390/genes15010119

**Published:** 2024-01-18

**Authors:** Andrea Hadjipanteli, Athina Theodosiou, Ioannis Papaevripidou, Paola Evangelidou, Angelos Alexandrou, Nicole Salameh, Ioannis Kallikas, Kyriakos Kakoullis, Sofia Frakala, Christina Oxinou, Andreas Marnerides, Ludmila Kousoulidou, Violetta C. Anastasiadou, Carolina Sismani

**Affiliations:** 1 The Cyprus Institute of Neurology and Genetics, Cytogenetics and Genomics, 2371 Nicosia, Cyprus; andreah@cing.ac.cy (A.H.);; 2 AAK Ultrasound and Fetal Medicine Centre, 2025 Nicosia, Cyprus; 3 Apollonion Private Hospital, 2054 Nicosia, Cyprus; 4 Ledra Clinic, 1060 Nicosia, Cyprus; 5 Christina Oxinou Histopathology/Cytology Laboratory, 1065 Nicosia, Cyprus; 6 Guy’s and St Thomas’ NHS Foundation Trust, London SE1 7EH, UK; 7 Archbishop Makarios III Hospital, 2012 Nicosia, Cyprus

**Keywords:** voltage-gated sodium channels, whole-exome sequencing, *SCN2A*, *SCN4A*, epileptic encephalopathy, severe fetal congenital myopathy 22B

## Abstract

Voltage-gated sodium channels (VGSCs) are responsible for the initiation and propagation of action potentials in the brain and muscle. Pathogenic variants in genes encoding VGSCs have been associated with severe disorders including epileptic encephalopathies and congenital myopathies. In this study, we identified pathogenic variants in genes encoding the α subunit of VGSCs in the fetuses of two unrelated families with the use of trio-based whole exome sequencing, as part of a larger cohort study. Sanger sequencing was performed for variant confirmation as well as parental phasing. The fetus of the first family carried a known de novo heterozygous missense variant in the *SCN2A* gene (NM_001040143.2:c.751G>A p.(Val251Ile)) and presented intrauterine growth retardation, hand clenching and ventriculomegaly. Neonatally, the proband also exhibited refractory epilepsy, spasms and MRI abnormalities. The fetus of the second family was a compound heterozygote for two parentally inherited novel missense variants in the *SCN4A* gene (NM_000334.4:c.4340T>C, p.(Phe1447Ser), NM_000334.4:c.3798G>C, p.(Glu1266Asp)) and presented a severe prenatal phenotype including talipes, fetal hypokinesia, hypoplastic lungs, polyhydramnios, ear abnormalities and others. Both probands died soon after birth. In a subsequent pregnancy of the latter family, the fetus was also a compound heterozygote for the same parentally inherited variants. This pregnancy was terminated due to multiple ultrasound abnormalities similar to the first pregnancy. Our results suggest a potentially crucial role of the VGSC gene family in fetal development and early lethality.

## 1. Introduction

Voltage-gated sodium channels (VGSCs) are responsible for the initiation and propagation of action potentials in the brain, skeletal muscle and heart [1]. Genes encoding VGSCs are amongst the most highly conserved genes in the human genome, which reflects their importance for normal human development and function [1]. Although this gene family shares high sequence homology, the proteins they encode have diverged in function over time and are expressed in different tissues. Therefore, pathogenic variants in this gene family can affect multiple organ systems [2] and cause epilepsy, heart defects, skeletal muscle disorders and various other abnormalities [3,4,5]. This gene family includes, among others, sodium voltage-gated channel α subunit 2 (*SCN2A*), which encodes the α subunit of NaV1.2 mainly expressed in the central nervous system [3], and sodium voltage-gated channel α subunit 4 (*SCN4A*), which encodes the α subunit of NaV1.4 and is predominantly expressed in skeletal muscle including fetal and neonatal [5].

Genetic disorders caused by the impaired function of VGSCs are called sodium channelopathies. Despite being characterized by a well-defined set of clinical features postnatally, sodium channelopathies can be difficult to diagnose due to their heterogeneous presentation. Sodium channelopathies have been associated with epileptic seizures, muscle stiffness, paralysis and cardiac structural abnormalities among others, depending on the affected gene. Clinical presentation can also vary in severity depending on the effect of the abnormality on the channel function. Successful diagnosis for genetic disorders such as sodium channelopathies is even more challenging prenatally. Advances in ultrasonographic imaging have permitted the visualization of previously undetectable fetal abnormalities leading to the identification of fetal abnormalities in approximately 2–5% of pregnancies [6,7]. Despite these advances, however, the literature lacks prenatal phenotypic descriptions linked to a wide range of Mendelian disorders including sodium channelopathies, creating challenges in prenatal diagnosis. As a result, fetal structural anomalies identified on ultrasound are often not suggestive of a particular disorder [7]. In addition, the identification and reporting of low-specificity characteristics (e.g., talipes and polyhydramnios) are often not helpful for the identification of a specific genetic disorder, since these characteristics are associated with various genetic abnormalities. Therefore, reaching a diagnosis in these cases is challenging and often unsuccessful. In other cases, certain phenotypes, which are characteristic of particular genetic disorders such as epilepsy, cannot be assessed in utero while other features may only be detectable at specific developmental stages, usually later in pregnancy [6,7,8,9]. The lack of knowledge regarding prenatal phenotypic abnormalities in the literature also hinders the identification of disorders where the prenatal phenotype differs from the known neonatal clinical picture, which is often the case [7,10]. It is also important to note that phenotypic abnormalities can develop as well as resolve at different gestational stages; therefore, the monitoring of fetal structural abnormalities throughout pregnancy can be informative in regard to the associated genetic disorder [11]. Generally, insufficient and incomplete reporting of prenatal manifestations in the literature is hampering the process of prenatal diagnosis and the identification of the genetic cause of a pregnancy loss. Studies have highlighted the need for variant databases that include prenatal phenotypes, which will assist prenatal diagnosis by creating accurate genotype–phenotype correlations [8]. Detailed and extensive reporting of prenatal clinical characteristics is highly important to expand the existing prenatal phenotypes associated with disorders such as sodium channelopathies. This will increase our understanding regarding genes crucial for human development and will assist diagnosis, and permit early interventions where applicable. 

In the past, the gold standard for the genetic investigation of abnormal fetal phenotypes and pregnancy loss was karyotype analysis, which is used to detect large-scale structural and numerical chromosomal aberrations of >5 Mb. Genomic microarray analysis is also routinely used in the prenatal setting to identify copy number variants (CNVs) as small as 30 kb [12,13]. Although this technique offers a higher resolution than chromosomal analysis, it is only capable of detecting unbalanced events [13] in the fetal genome. These tests combined identify a causative genetic abnormality in up to 40% of cases presenting fetal abnormalities, while the rest do not typically receive a genetic diagnosis [14]. However, pregnancy loss and abnormal fetal phenotypes can also arise as a result of smaller or balanced genetic abnormalities such as indels and single nucleotide variants (SNVs). Recently developed Next Generation Sequencing (NGS) is capable of detecting such abnormalities [15]. NGS has revolutionized sequencing and has proven to be a valuable tool for postnatal diagnosis as it allows the detection of genetic abnormalities down to a single base pair [16]. More specifically, studies have shown that the use of whole exome sequencing (WES) postnatally can generate a diagnostic yield of 20–80% based on the reason for referral [7,17]. However, challenges in the interpretation of single-nucleotide variants (SNVs) have delayed the incorporation of WES in prenatal diagnostic pipelines. Regardless, relevant research regarding its potential application for fetal genetic investigation has been carried out. According to studies, the prenatal diagnostic yield of WES can reach 6.2–57.1% depending on the affected organ system, the number of affected organ systems, single vs. trio sequencing and the extent of prenatal phenotypic description [11]. Studies including fetuses with any ultrasound anomaly tend to score a lower diagnostic yield than studies including fetuses with highly specific and extensive phenotypic descriptions [7]. This is because an incomplete prenatal clinical picture creates difficulties in the identification and interpretation of WES variants [18]. Overall, WES can improve diagnostic rates of cases with extensive fetal structural abnormalities where previous testing, including karyotype and array-CGH, is unrevealing. It can thus assist decision making regarding the pregnancy, newborn care as well as calculate the chance of recurrency in future pregnancies of the family. In turn, this can decrease the fetal/newborn death rate and healthcare costs, as well as the emotional turmoil of the couple [14]. Recent studies also suggest that upon the completion of WES, multidisciplinary consensuses can assist prenatal diagnosis and make recommendations regarding the management of fetal disorders to improve fetal prognosis [8]. The use of WES in combination with the detailed reporting of prenatal manifestations and management from multidisciplinary consensuses can create new genotype in utero–phenotype associations, improve our understanding of fetal genetic disorders and embryonic lethal variants [6,9] and ultimately assist the incorporation of NGS in the prenatal diagnostic setting. 

As part of a larger cohort study, we collected prenatal samples from fetuses presenting abnormal sonographic findings and prenatal/neonatal death and employed trio-based WES to uncover pathogenic variants related to the phenotype of interest. Here, we present the fetuses of two unrelated families from this cohort study, where the identified variants were located in genes encoding the α subunit of the VGSCs. This study aims to assess the role of VGSCs in fetal development and survival and to enrich the prenatal clinical picture related to *SCN2A* and *SCN4A* by reporting phenotypic manifestations that, to our knowledge, had not been previously reported. 

## 2. Materials and Methods

### 2.1. Patients and Samples

As part of a larger cohort study, prenatal samples including amniotic fluid, chorionic villi or skin biopsies were collected from fetuses with abnormal ultrasound findings and/or prenatal death. These samples were referred to the Cytogenetics and Genomics Department of the Cyprus Institute of Neurology and Genetics for diagnostic genetic analysis including karyotype and array-CGH. Referred samples with a normal karyotype and array-CGH result were then selected along with parental samples to undergo prenatal research trio-based WES to identify causative variants relevant to the phenotype of interest. Out of 33 families that were enrolled in this cohort study, 2 presented clinical abnormalities consistent with sodium channelopathies and aberrations in genes encoding VGSCs. This study, including the corresponding consent forms, was approved by the Cyprus National Bioethics Committee (ΕΕΒΚ/ΕΠ/2020/38) and informed consent was obtained from all participating families, allowing the use of their biological samples in this study and associated publications. All clinical features were described using Human Phenotype Ontology (HPO) terms [19].

#### 2.1.1. Family 1

The proband in family 1 was a female fetus of Greek-Cypriot ancestry. The amniotic sample for DNA isolation was collected at 22 weeks of gestation and was referred for diagnostic genetic analysis using karyotype and array-CGH due to multiple phenotypic abnormalities identified on sonographic imaging prenatally, including hand clenching (HP:0001188), ventriculomegaly (HP:0002119), aplasia of the gallbladder (HP:0011466), pericardial effusion (HP:0001698) and intrauterine growth retardation (HP:0001511). Both karyotype and array-CGH had a normal result. The couple continued with the pregnancy and the trio was enrolled into our research study. Postnatal patient follow-up also revealed epileptic spasms (HP:0011097), skeletal muscle atrophy (HP:0003202) and refractory status epilepticus (HP:0032867). Polymicrogyria (HP:0002126)/lissencephaly (HP:0001339) was also identified on MRI. The baby died neonatally. Both parents were apparently healthy. At the time of birth, the mother was 28 years old and the father was 36 years old. 

#### 2.1.2. Family 2

The proband in family 2 was a male fetus of Greek-Cypriot ancestry. Amniotic fluid was collected for DNA isolation at 31.4 weeks of gestation and the fetus was referred for diagnostic genetic analysis using karyotype and array-CGH with a suspicion of Pallister–Killian syndrome. The fetus presented decreased fetal movement (HP:0001558), talipes equinovarus of the left foot (HP:0001762), talipes calcaneovarus of the right foot (HP:0001884) as well as polyhydramnios (HP: 0001561), which were identified via ultrasound prenatally. Both karyotype and array-CGH had a normal result. Pallister-Killian syndrome was excluded and the couple continued with the pregnancy. At birth, the proband had a body weight of 2410 g, head circumference of 35 cm and length of 51 cm. The baby died as soon as the umbilical cord was cut at delivery, which took place at 36 weeks. Both parents were apparently healthy. At the time of birth, the mother was 33 years old and the father was 35 years old. In a subsequent pregnancy of the couple, DNA was isolated from amniotic fluid, which was collected at 16.6 weeks of gestation. The second fetus was female and was referred for genetic investigation with Sanger sequencing to confirm the presence of findings previously identified in the proband, in the absence of any phenotypic abnormalities. The pregnancy was later terminated at 24 weeks of gestation due to the identification of severe structural abnormalities at 22 weeks.

Pediatric post-mortem examination was carried out on both the baby and the fetus. In the case of the first baby, it confirmed the presence of talipes equinovarus of the left foot and talipes calcaneovarus of the right foot and also revealed single transverse palmar crease (HP:0000954), anteverted nares (HP:0000463), pulmonary hypoplasia (HP:0002089) and narrow thorax (HP:0000774). In the second fetus, the fetopsy identified talipes equinovarus of the left foot (HP:0001762), talipes equinovalgus of the right foot (HP:0001772), posteriorly rotated ears (HP:0000358) and micrognathia (HP:0000347). Most importantly, the post-mortem examinations revealed the presence of findings, which were identical in both the baby and the fetus. The findings were low-set ears (HP:0000369), high arched palate (HP:0000218), bilateral diaphragmatic eventration (HP:0009110), disproportionality of the head with the anterior–posterior axis of the skull vault being disproportionately greater than the transverse axis and the head appearing large in relation to the trunk and limbs, as well as reduced muscle bulk of the limbs and the psoas muscles with myopathic-type histologic changes on a microscopic examination of various skeletal muscles.

### 2.2. Genetic Investigation

Fetal DNA was isolated from amniotic fluid using the QIAGEN QIAamp DNA Mini Kit. Parental DNA was also isolated from peripheral blood using the QIAGEN QIAamp DNA Blood Midi Kit according to the manufacturer’s instructions.

Library preparation was performed using Illumina’s DNA Prep with Enrichment kit and exome sequencing was carried out using Illumina NextSeq 500/550 High Output Kit v2.5 on the Illumina NextSeq 500 System as per the manufacturer’s protocol. After completion of the WES run, trio check was carried out using Peddy software package v0.4.2 on a Linux machine to ensure genetic relatedness between the fetus and parents. Fastq files were loaded on VarSome Clinical Saphetor SA platform [20] for alignment (hg19 reference genome), variant calling and annotation. Predicted variants were filtered based on minor allele frequency (MAF), inheritance mode, coverage (>8 at the specific locus) and genomic location (variants targeted in coding and splicing regions ±10 base pairs). The final verdict regarding the pathogenicity of each variant was assessed based on the recommendations of the ACGS 2020 guidelines [21], ClinGen sequence variant interpretation working group (30) and was assisted by the automatic scoring ACMG classification of VarSome Clinical (v10.1) and VarSome (v11.3.10). Variants classified as pathogenic, likely pathogenic and variants of unknown significance (VUS), where a strong rule suggesting pathogenicity was fired based on ACGS, were prioritized as they are more likely to have an effect on the protein. Conservation scores were also taken into account since evolutionarily conserved regions of the genome tend to be of high importance for cellular function. Lastly, the phenotypic features of our probands were compared against other reported cases in the literature carrying either the same variant or different variants within the same gene. Variants were reported in cases where the proband’s phenotype matched the phenotype observed in other patients carrying variants in the same gene. 

To validate trio-based WES results, Bidirectional Sanger sequencing was employed, using specifically designed primers (Metabion, Munich, Germany) through the Primer3 web interphase tool flanking the mutation site. Applied Biosystems™ BigDye™ Terminator v1.1 Cycle Sequencing Kit (ThermoFisher Scientific, Waltham, Massachusetts, USA) and Affymetrix ExoSAP-IT^®^† (Fisher Scientific, Pittsburgh, PA, USA) were used for this procedure. 

## 3. Results

### 3.1. Family 1

Trio-based WES analysis revealed the presence of a heterozygous de novo, single nucleotide, missense variant in exon 8 of the *SCN2A* gene, OMIM #182390 [22] (NM_001040143.2:c.751G>A, p.(Val251Ile); NC_000002.11:g.166166886G>A; rs1057519528) in the proband (Supplementary Material Figure S1). The change was classified as pathogenic based on previously mentioned guidelines and applying the following rules: PS2—strong; PS4—moderate; PM1—moderate; PP3—moderate; and PM2—supporting. The variant is located in the transmembrane region of the S5 pore module in DI, which is a mutational hot-spot domain. Based on VarSome, Franklin by genoox and UCSC genome browser [23], a low rate of benign variation has been identified in this region. This variant is predicted to be pathogenic based on multiple Meta prediction scores including REVEL, MetaSVM and BayesDel noAF and was absent from gnomAD population databases. Conservation prediction tools were employed to assess the evolutionary conservation of the transmembrane region that the variant was located in. Tools such as PhyloP100way (9.818) have predicted the region to be highly conserved. The variant was reported twice in ClinVar as pathogenic in one Developmental and Epileptic Encephalopathy 11 (DEE11) patient (RCV001329203) (SCV001520577.1) and in one Epileptic Encephalopathy patient (RCV000416970) (SCV000494509.1) [24]. 

Sanger sequencing confirmed the presence of the variant in heterozygous state in the proband and noncarrier status in both parents, thus also confirming the de novo origin of the variant (Supplementary Material Figure S2).

### 3.2. Family 2

Two variants were identified in gene *SCN4A*, OMIM #603967 [22] in a compound heterozygous state in the proband using trio-based WES analysis (Supplementary Material Figures S3 and S4). The paternally inherited variant was a missense SNV (NM_000334.4:c.4340T>C, p.(Phe1447Ser); NC_000017.10:g.32019302A>G), classified as likely pathogenic based on rules: PP3—strong; PM1—moderate; PP3—moderate; and PM2—supporting. The variant is located on the S4 of DIV and is predicted to be pathogenic based on multiple Meta prediction scores including REVEL, MetaSVM and BayesDel noAF. The variant scored 9.075 in PhyloP100way scale, which shows that the region is evolutionarily highly conserved. It is located in a mutational hot-spot and is absent from gnomAD population databases and considered to be novel as it has not been previously reported in any frequency databases. A nearby maternally inherited missense SNV was also identified in *SCN4A* gene (NM_000334.4:c.3798G>C, p.(Glu1266Asp); NC_000017.10:g.62022147C>G). The variant was classified as likely pathogenic based on PP3—moderate; PM2—supporting; and PP4—supporting. This variant is located on the extracellular pore loop domain of DIII and it is absent from gnomAD population databases. Although this variant was absent from frequency databases, a cohort study has reported it in homozygosity in a patient with myopathic changes [25].

Sanger sequencing confirmed the presence of both variants in the proband in a compound heterozygous state and confirmed the carrier status of each variant in the parents (Supplementary Material Figures S5 and S6). The second fetus had been referred prenatally for Sanger sequencing, which confirmed the presence of both variants in a compound heterozygous state in the absence of any ultrasound findings. This pregnancy was later terminated due to the development of phenotypic abnormalities identical to the first fetus. 

## 4. Discussion

**Figure 1:**
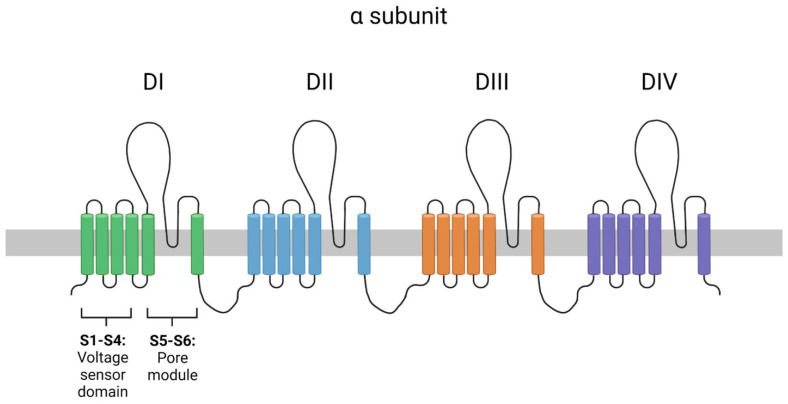
Topological scheme of the α subunit of VGSCs. DI-DIV represent the four homologous domains. Each domain is composed of six α-helical segments. Segments 5 and 6 form the ion pore and segments 1–4 form the voltage sensor domain. (Created with BioRender.com, accessed on the 17 December 2023).

VGSCs are heteromeric, membrane-associated proteins, composed of one α and one to four β subunits. The α subunit is made of four homologous domains (DI-DIV). Each domain is made of six transmembrane α helical segments (S1–S6). Segments 5–6 form a highly selective Na+ pore with a pore loop located between S5 and S6, while segments 1–4 form the voltage-sensing domain that regulates the opening and closing of the pore [2] (Figure 1). Mammalian NaV channels have a selectivity filter, which is composed of a single residue in each of the four pore-loops of the four functional domains. These residues create a ring composed of Glu in DI, Asp in DII, Lys in DIII and Ala in DIV (DEKA), which functions as a selectivity filter. The β subunits are responsible for the trafficking and electrophysiological properties of the α subunit [1,2]. Studies have shown that particular impairments in NaV function, especially complete LOF and particular GOF, are incompatible with life while other impairments can cause severe phenotypic abnormalities. These findings along with the neonatal deaths observed in the cases we are presenting highlight the importance of these channels in normal fetal development [26,27]. The reporting of variants resulting in NaV impairments along with relevant phenotypes is therefore of utmost importance for fetal prognosis.

In this study, we identified variants in two genes encoding the α subunit of VGSCs in fetuses of two unrelated families with abnormal sonographic findings. 

### 4.1. Family 1

A heterozygous missense de novo SNV in *SCN2A* was identified in a fetus presenting multiple congenital abnormalities, severe neonatal epileptic manifestations and early lethality. The variant has a pathogenic verdict based on ACGS 2020 rules, suggesting a potentially deleterious effect on the NaV1.2 channel. It is located on the transmembrane S5 of DI of NaV1.2, which is an evolutionarily conserved region. Therefore, a variant in this domain can have detrimental effects on the channel function, stability and trafficking, resulting in severe phenotypes and/or lethality [1]. 

NaV1.2 is the principal VGSC in excitatory pyramidal neurons, mainly expressed in axons and nerve terminals [28,29]. Variants in *SCN2A* have previously been identified in patients with mild clinical manifestations; however, as more variants are uncovered, they are increasingly being associated with various epileptic disorders and more severe phenotypic abnormalities [30]. Variants within this gene were first reported in patients with benign familial neonatal-infantile epilepsies (BFNIEs) [3]. The phenotypic spectrum associated with *SCN2A* has largely expanded since, as variants have also been reported in patients with epilepsy of infancy with migrating focal seizures (EIMFS), infantile spasms, episodic ataxia 9, Ohtahara syndrome, developmental and epileptic encephalopathy 11 (DEE11) and other non-specific epileptic phenotypes. *SCN2A* variants have also been found in patients with autistic features in the absence of epilepsy [30]. 

Gain-of-function (GOF) variants typically cause early seizure onset (≤3 months of life) and have been linked with infantile onset epileptic encephalopathies, Ohtahara syndrome, West syndrome and BFNIS [29]. GOF variants can result in a persistent increase in Na+ influx and a decrease in the refractory time after an action potential, causing the hyperexcitability of neurons resulting in epileptic seizures [31]. The epileptic severity varies depending on the nature of the GOF variant and its electrophysiological effects, which explains why GOF NaV1.2 variants can cause both mild BFNIS and severe infantile epileptic encephalopathies [32,33]. Heterogeneity can even be observed in patients carrying the same variant, likely due to the effect of other genetic modifiers [3]. We propose that the epileptic phenotype observed in our proband neonatally was a result of the aforementioned variant, which causes a GOF in NaV1.2. 

Several studies have shown that most epilepsy-related missense variants identified in *SCN2A* usually occur de novo [2,34] as in the case of family 1, which enhances the pathogenicity of the variant. The same variant was also previously reported as pathogenic/likely pathogenic twice in ClinVar in DEE11 and epileptic encephalopathy patients, respectively [24]). Postnatally, these patients typically present refractory epilepsy and movement disorders, while some patients also present highly variable brain magnetic resonance imaging (MRI) abnormalities [3,34,35]. The current literature is in concordance with our proband’s findings, which included refractory epilepsy, spasms and MRI abnormalities such as polymicrogyria/lissencephaly postnatally [3,28,31]. It is important to note that phenotypic characteristics related to *SCN2A* GOF variants such as refractory epilepsy and spasms have a neonatal onset and cannot be assessed in utero. Therefore, the identification of phenotypes such as ventriculomegaly is important as it could be used as an indication for an *SCN2A* channelopathy prenatally. Following the identification of ventriculomegaly, complementary prenatal MRI could also be employed to reveal brain abnormalities characteristic of specific disorders such as *SCN2A* channelopathies [36]. Certain cardiac abnormalities have also been sporadically reported in *SCN2A* channelopathies. In this study, we expand the findings relating to the cardiovascular system by reporting pericardial effusion. The aforementioned characteristics that could potentially refer to *SCN2A* channelopathies, should prompt the use of prenatal WES for the identification of the pathogenic variants and confirmation of diagnosis [37].

The current case enriches the existing prenatal phenotypic spectrum suggestive of *SCN2A* channelopathies, which are usually apparent postnatally with the development of seizures and neurocognitive abnormalities [14]. In this case, we identified prenatal phenotypes such as pericardial effusion, IUGR, hand clenching and aplasia of the gallbladder, which differ considerably from those typically observed in the newborn. Early identification of these prenatal characteristics should encourage the use of prenatal WES to identify the genetic etiology. WES results will guide the couple in decision making regarding the pregnancy and the referring physicians regarding newborn management [14]. The phenotypes identified in our proband and other similar cases could be used as markers in the future when more cases are uncovered. Nevertheless, we cannot exclude the possibility of a second, unidentified variant being responsible for some of the proband’s prenatal phenotypes that do not fit the expected clinical presentation, including aplasia of the gallbladder, pericardial effusion and ventriculomegaly. 

### 4.2. Family 2

Trio-based WES analysis revealed the presence of two missense SNVs in *SCN4A* of the proband and the second fetus, each inherited from a different parent, resulting in a compound heterozygous state. Both the paternally and maternally inherited variants were classified as likely pathogenic based on the ACGS 2020 rules, suggesting a potentially high impact of both variants resulting in a destructive effect on NaV1.4.

**Figure 2:**
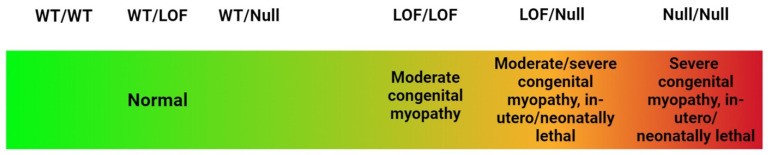
Diagram illustrating the relationship between Wild Type (WT), LOF and null *SCN4A* variants and the spectrum of disorders and phenotypic features associated with them. (Created with BioRender.com, accessed on the 17 December 2023).

*SCN4A* is most commonly associated with dominant GOF variants, which are a well-established cause of various sodium channelopathies such as myotonia and hyperkalemic periodic paralysis [26]. GOF of NaV1.4 causes muscle stiffness, involuntary muscle contractions/spasms, paralysis attacks and neonatal hypotonia. and Feeding and respiratory difficulties have also been reported [38]. Recessive LOF variants within this gene are much rarer and have only been identified in a small number of cases [39]. Heterozygous *SCN4A* LOF variants are not typically sufficient to cause an abnormal phenotype [26,38], which is consistent with the phenotypically normal carrier parents in family 2. However, homozygous LOF variants can cause a wide spectrum of phenotypic abnormalities, with heterogeneous presentation even within families, depending on the nature of the variant [39] (Figure 2).

Homozygous LOF variants were first identified in congenital myasthenic syndrome patients. These patients usually present ptosis, bulbar weakness, respiratory difficulties and prolonged episodes of weakness. These variants were also later identified in patients with classical congenital myopathy [26]. More recently, LOF variants were also found in patients with a new form of *SCN4A*-related congenital myopathy named severe fetal congenital myopathy 22B, (OMIM #603967) [26]. Congenital myopathies are a group of disorders with genetic and clinical heterogeneity ranging from mild classical congenital myopathy phenotype with a normal life expectancy to severe fetal hypokinesia and early lethality [26,38].

A small cohort study presented a few patients with severe fetal congenital myopathy 22B carrying homozygous and compound heterozygous LOF variants in *SCN4A*. They proposed that the combination of two *SCN4A* LOF variants, where at least one of them is a null variant, constricts the amplitude of action potentials and therefore impairs normal muscle force [26]. The clinical features associated with severe fetal congenital myopathy 22B differed from the ones observed in sodium channelopathies caused by GOF variants and the few cases of classical congenital myopathy that were previously reported [26]. Prenatally, all patients in this cohort presented in utero/neonatal muscle weakness leading to fetal hypokinesia, while a number of severely affected cases also presented polyhydramnios similar to our proband. Severe clinical presentation also includes features related to reduced fetal movement such as limb contractures and talipes [26]. Severe hypokinesia of the fetus is also associated with a narrow thorax, hypoplastic lungs and traits suggestive of hydrops such as polyhydramnios, which are identified from the 19th week of gestation onwards. This is in agreement with the fetuses we are presenting that only developed phenotypic abnormalities near the end of the second trimester. Muscle hypoplasia, plagiocephaly/frontal bossing, ear abnormalities and a high arched palate have also been observed in these patients, among other characteristics [26]. Severe clinical presentation including in utero/neonatal death was observed in patients carrying homozygous null variants in *SCN4A* and in some patients with compound heterozygous *SCN4A* variants, carrying combinations of complete and partial LOF in NaV1.4. These results suggest that complete and, in some cases, partial abolition of NaV1.4 function in muscle is incompatible with life, highlighting the importance of NaV1.4 in development and survival.

The phenotype of both fetuses in the family we are presenting is in concordance with the findings of this cohort, suggesting that a potential compound heterozygous loss-of-function variant in *SCN4A* was responsible for their clinical picture. In this study, we also present phenotypic abnormalities that were absent from the cohort patients, including bilateral diaphragmatic eventration, single transverse palmar crease and anteverted nares, potentially enriching the phenotypic spectrum associated with LOF *SCN4A* variants. Reaching the prenatal diagnosis of severe fetal congenital myopathy 22B can be challenging since muscular abnormalities/pathology are difficult to assess in utero. It is therefore important to identify features such as hypotonia, fetal hypokinesia and talipes, which could be suggestive of this disorder. These findings indirectly suggest the presence of a musculoskeletal disorder, which can be confirmed using genetic analysis. 

The phenylalanine at position 1447 (paternally inherited variant) is one residue upstream of the first Arg in the conserved sequence R-x-x-R-x-x-R-x-x-R-x-x-R-x-x-K (R-V-I-R-L-A-R-I-G-R-V-L-R-L-I-R-G-A-K) in the voltage sensor S4 of DIV. A variant at this position could potentially affect the gating properties of channel inactivation resulting in reduced channel availability [5,39]. The glutamate at position 1266 (maternally inherited variant) is highly conserved in all isoforms of the human voltage-gated sodium channels in the extracellular part of the pore loop in DIII. The variant is located 22 residues downstream from the critical K (Lys) in the DEKA selectivity filter; therefore, the channel permeability will likely not be affected. However, the variant could potentially cause defects in channel selectivity, a loss of charge conservation and thus could reduce the channel conductance, since studies have shown that K in the DEKA selectivity filter has an active role in channel conductance [40].

Although functional investigation is required to confirm the pathogenicity of the identified variants, the phenotypic features identified in both fetuses of the family prenatally were in concordance with the ones described in the aforementioned cohort. Pediatric post-mortem examination further confirmed the diagnosis of severe fetal congenital myopathy 22B, based on the phenotypic features identified in the cohort. In addition, the maternally inherited variant we identified had previously been reported in homozygosity, in a patient with facial hypomimia, limb girdle weakness and fatigability [25] enhancing its pathogenic prediction. Thus, we propose that the phenotype observed in both fetuses of this family is potentially the result of severe fetal congenital myopathy 22B, caused by compound heterozygous LOF variants in *SCN4A*. Overall, the identification of causative variants using WES has not only enriched the phenotypic spectrum associated with severe fetal congenital myopathy 22B but has also provided the couple with the necessary information for informed decision making regarding their future pregnancies and family planning. 

## 5. Conclusions

In agreement with other studies, we highlight the importance of prenatal WES in fetuses with a normal karyotype and array-CGH result, especially in cases presenting skeletal and multisystem anomalies [8,10]. This study has contributed to enriching knowledge regarding the development of fetuses carrying variants in genes encoding VGSCs. We also highlight the importance of a detailed prenatal clinical description as well as the use of post-mortem examinations, which can reveal additional phenotypic characteristics, in patients undergoing WES. 

We have reported undocumented phenotypic features associated with *SCN2A* and *SCN4A* variants and two novel variants linked to severe fetal congenital myopathy 22B. The severe fetal sonographic anomalies and early lethality of our probands are consistent with other cases found in the literature, reinforcing the importance of VGSCs for normal fetal development. The use of WES leads to the reporting of new variants and new phenotypic features associated with VGSC genes, which is important for creating new genotype in utero–phenotype associations and assisting prenatal diagnosis. Therefore, reporting novel variants and undocumented clinical presentations related to the VGSC gene family may ultimately lead to new additions in preconception/prenatal diagnostic panels, facilitate genetic counselling and assist treatments and early interventions.

## Data Availability

Sequence variants found in the gene *SCN4A* are available on the ClinVar database (accession numbers: SCV004015158 and SCV004015159).

## Supplementary Materials

The following supporting information can be downloaded at: https://www.mdpi.com/article/10.3390/genes15010119/s1, Table S1: All rare variants of the fetus in family 1, Table S2: All rare variants of the fetus in family 2, Figure S1: IGV tracks showing the *SCN2A* variant in family 1, Figure S2: Electropherograms showing the *SCN2A* variant in family 1, Figure S3: IGV tracks showing the paternally inherited *SCN4A* variant in family 2, Figure S4: IGV tracks showing the maternally inherited *SCN4A* variant in family 2, Figure S5: Electropherograms showing the paternally inherited *SCN4A* variant in family 2, Figure S6: Electropherograms showing the maternally inherited *SCN4A* variant in family 2.
