# Supplementary material for: Sodium Channel Gene Variants in Fetuses with Abnormal Sonographic Findings: Expanding the Prenatal Phenotypic Spectrum of Sodium Channelopathies
Source: Genes (Basel). 2024 Jan 18;15(1):119. doi: 10.3390/genes15010119 (PMC10815715; doi:10.3390/genes15010119)
Supplement: Supplementary file 1 [file genes-15-00119-s001.zip › Supplementary Figures.pdf]

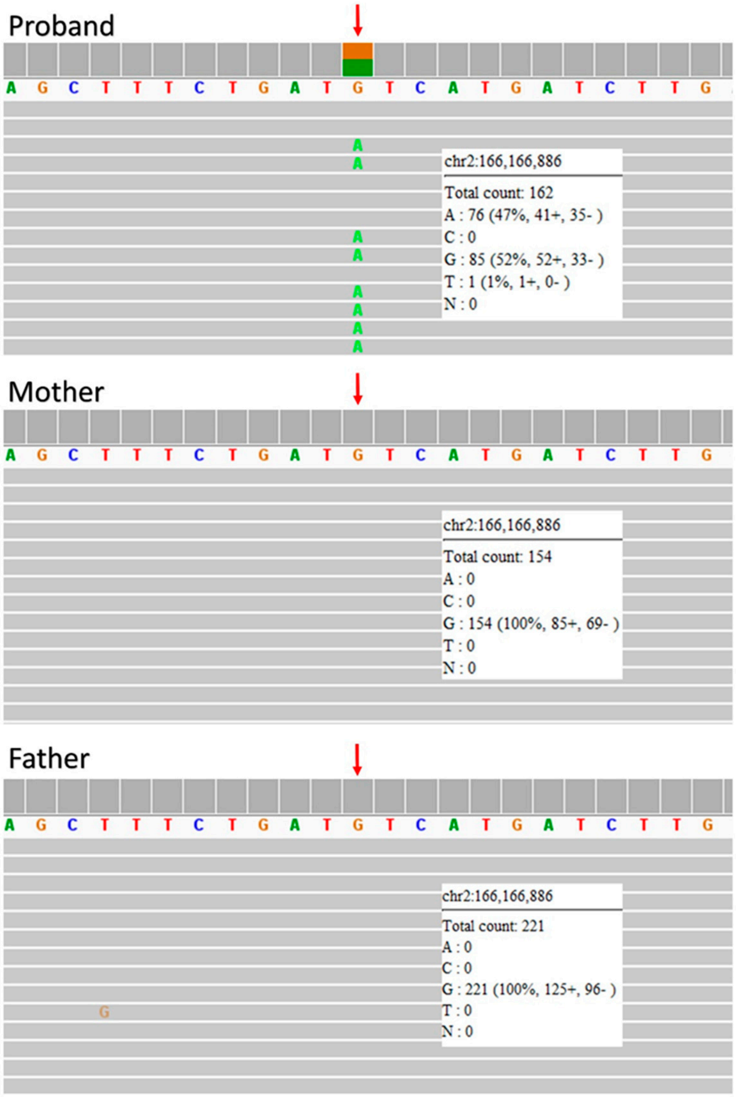

Figure S1. WES data aligned against hg19 reference genome, displayed on IGV tracks showing variant *SCN2A*:c.751G>A. The tracks represent the proband, mother and father in respective order where proband exhibits the variant in a de novo heterozygous manner, and both parents exhibit the wild type allele.

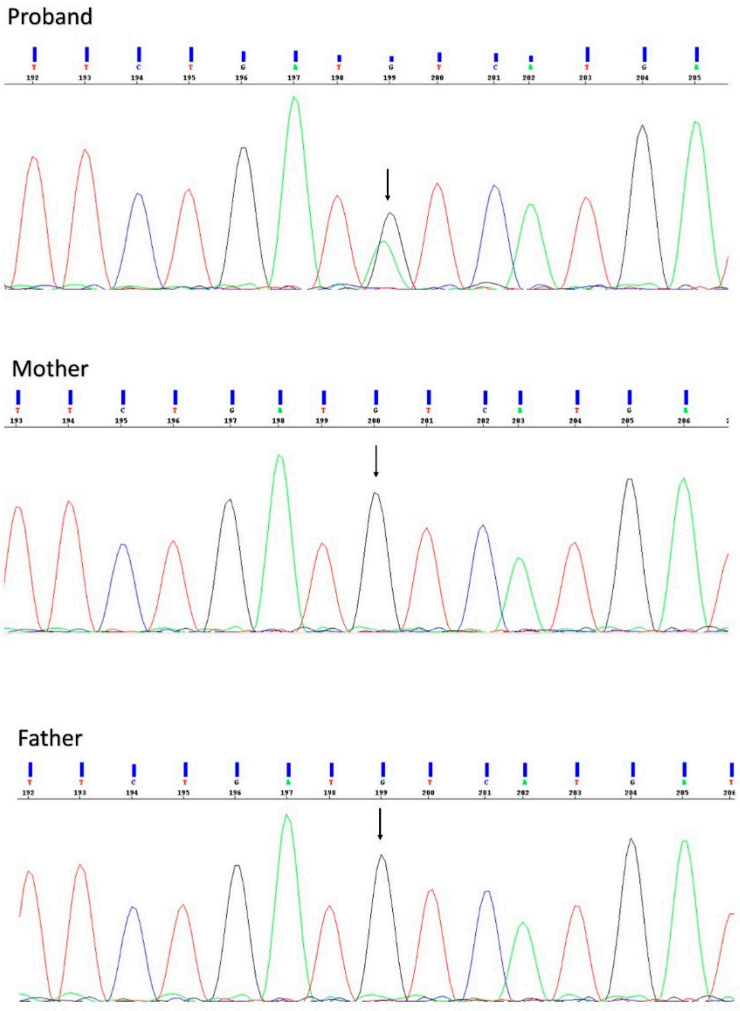

Figure S2. Sanger sequencing validation confirming the de novo origin of the variant *SCN2A*:c.751G>A in the proband, present in heterozygous state. Both parents are homozygous for the wild type (WT) allele.

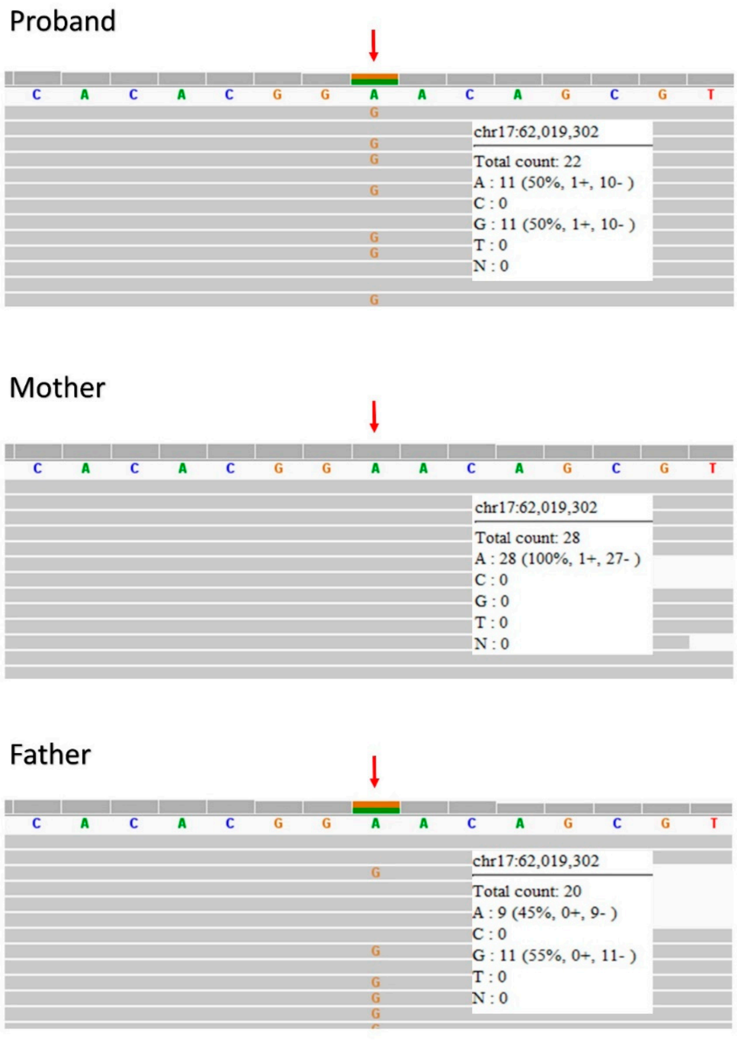

Figure S3. WES data aligned against hg19 reference genome, displayed on IGV tracks showing variant *SCN4A*:c.4340T>C in heterozygosity in the proband and father. The mother is homozygous for the wild type allele. The tracks represent proband, mother and father in respective order.

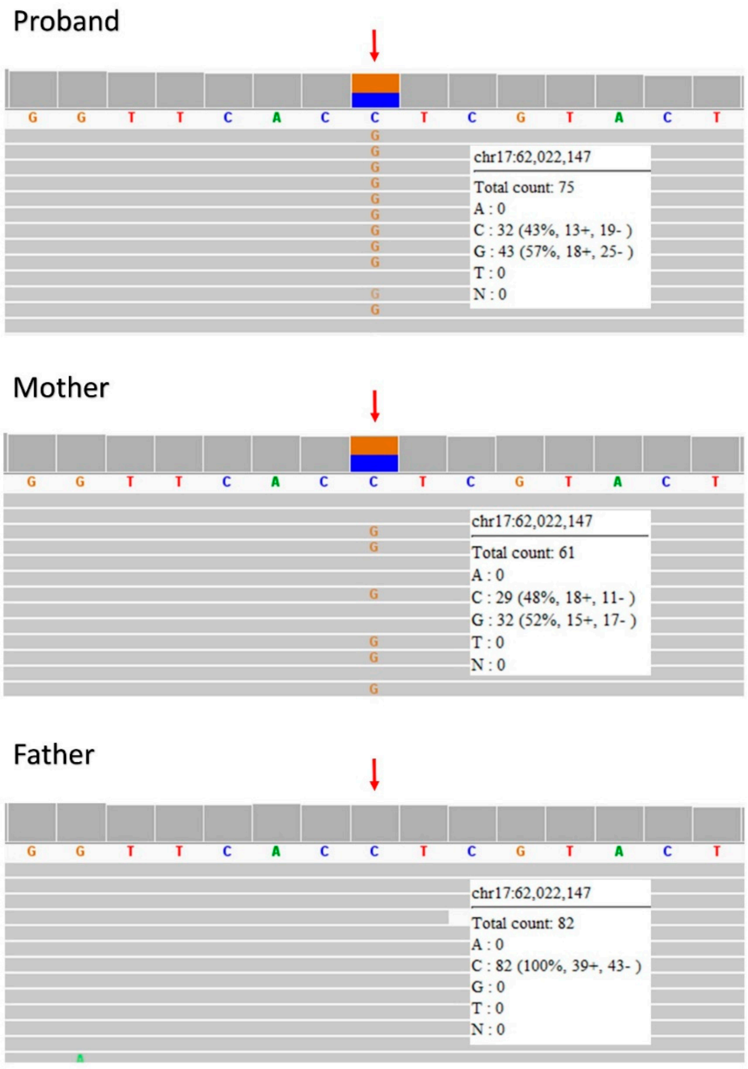

Figure S4. WES data aligned against hg19 reference genome, displayed on IGV tracks showing variant *SCN4A*:c.3798G>C in heterozygosity in the proband and mother. The father is homozygous for the wild type allele. The tracks represent proband, mother and father in respective order.

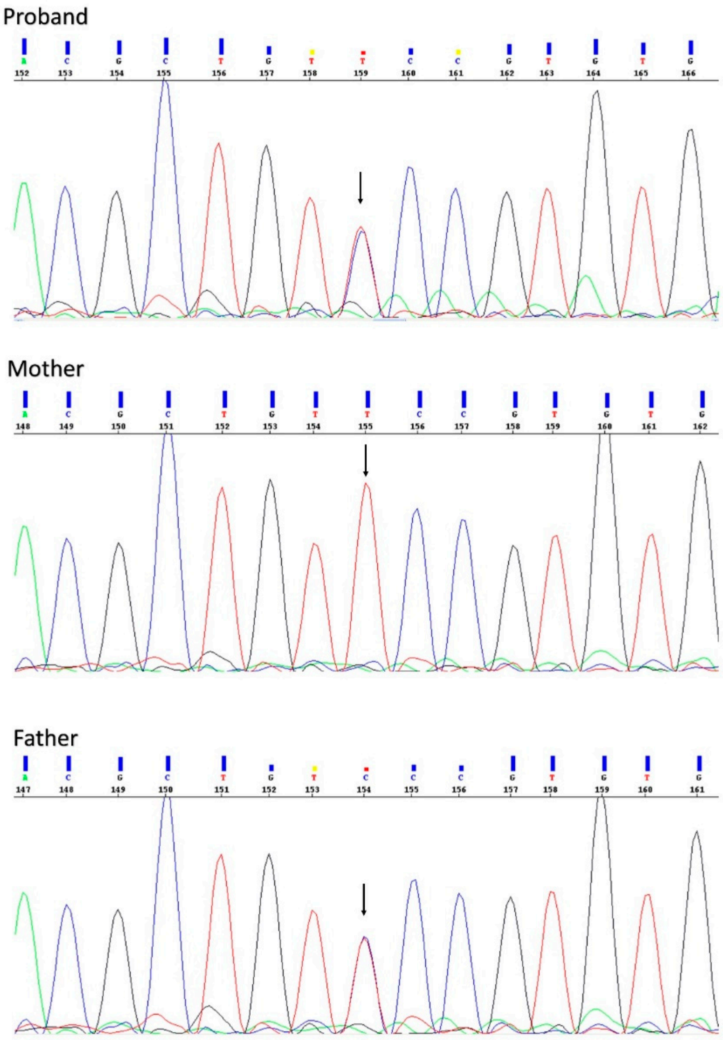

Figure S5. Sanger sequencing validation confirming the presence of *SCN4A*:c.4340T>C in heterozygosity in the proband and father. The mother is homozygous for the wild type allele.

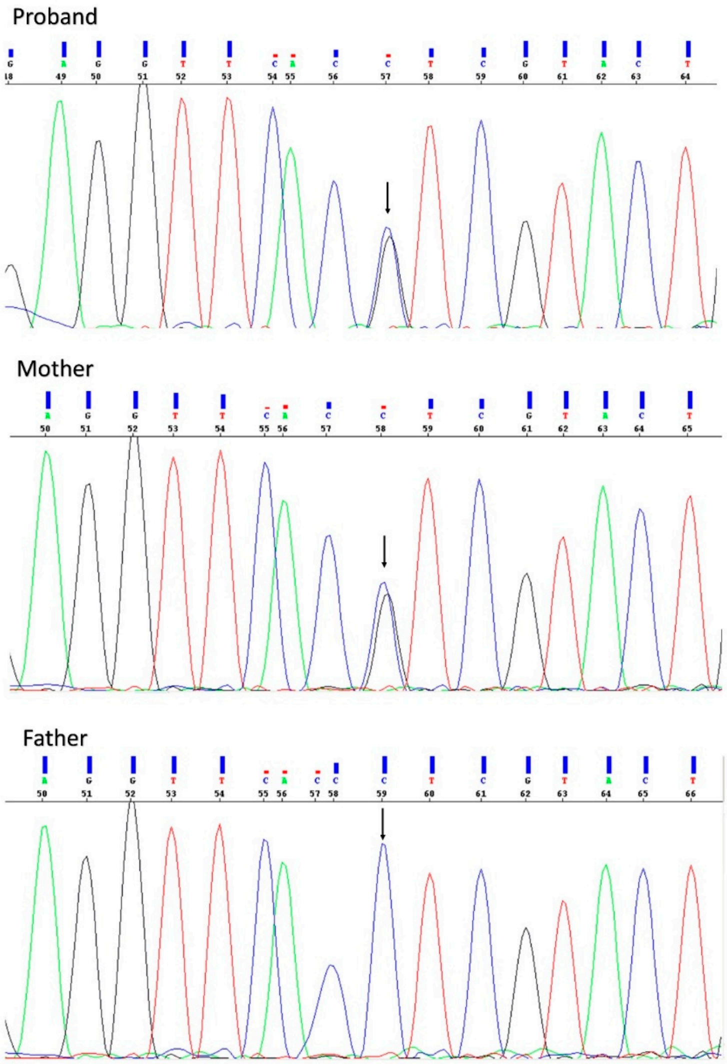

Figure S6. Sanger sequencing validation confirming the presence of *SCN4A*:c.3798G>C in heterozygosity in the proband and mother. The father is homozygous for the wild type allele.
